# Antineoplastic Effect of Lenvatinib and Vandetanib in Primary Anaplastic Thyroid Cancer Cells Obtained From Biopsy or Fine Needle Aspiration

**DOI:** 10.3389/fendo.2018.00764

**Published:** 2018-12-18

**Authors:** Silvia Martina Ferrari, Concettina La Motta, Giusy Elia, Francesca Ragusa, Ilaria Ruffilli, Luca Quattrini, Sabrina Rosaria Paparo, Simona Piaggi, Armando Patrizio, Salvatore Ulisse, Enke Baldini, Gabriele Materazzi, Poupak Fallahi, Alessandro Antonelli

**Affiliations:** ^1^Department of Clinical and Experimental Medicine, University of Pisa, Pisa, Italy; ^2^Department of Pharmacy, University of Pisa, Pisa, Italy; ^3^Department of Translational Research and of New Technologies in Medicine and Surgery, University of Pisa, Pisa, Italy; ^4^Department of Experimental Medicine, “Sapienza” University of Rome, Rome, Italy; ^5^Department of Surgical, Medical, Molecular Pathology and Critical Area, University of Pisa, Pisa, Italy

**Keywords:** anaplastic thyroid cancer, fine needle aspiration, lenvatinib, primary anaplastic thyroid cancer cells, tyrosine kinase inhibitors, vandetanib

## Abstract

Anaplastic thyroid carcinoma (ATC) is a malignant tumor of the thyroid gland, infrequent but with a very poor prognosis, as it rapidly causes death (mean survival of about 6 months). ATC treatment includes a multimodal protocol consisting of surgery, chemotherapy (doxorubicin and cisplatin), and hyperfractionated accelerated external beam radiotherapy (median patient survival of 10 months). For this reason, the identification of an effective systemic treatment for ATC would be a major advance in the management of this deadly thyroid cancer. The opportunity to test the sensitivity to different drugs of primary cells from ATC (pATC) cultures, obtained from each patients, could improve the effectiveness of the treatment. Then, the administration of inactive therapeutics could be avoided. Our aim is to investigate the antineoplastic effect of two tyrosine kinase inhibitors (TKIs; lenvatinib, vandetanib) in pATC obtained both from biopsy (biop-pATC), and from fine needle aspiration (FNA-pATC). The antiproliferative activity of lenvatinib and vandetanib was evaluated in 6 ATC patients, on biop-pATC, such as on FNA-pATC. A significant reduction of proliferation (obtained by WST-1 assay) vs. control was shown with lenvatinib and vandetanib in FNA-pATC, as well as in biop-pATC. The percentage of apoptosis in FNA-pATC, or biop-pATC, increased with both compounds dose-dependently. pATC cells from FNA, or biopsy, had a similar sensitivity to lenvatinib and vandetanib. In conclusion, primary cells (biop-pATC or FNA-pATC) have a similar sensitivity to TKIs, and lenvatinib and vandetanib are effective in reducing cell growth, increasing apoptosis in ATC. The possibility to test the sensitivity to different TKIs in each patient could open the way to personalized treatments, avoiding the administration of ineffective, and potentially dangerous, drugs.

## Introduction

Anaplastic thyroid carcinoma (ATC) is a malignant tumor of the thyroid gland, infrequent but with a very poor prognosis, as it rapidly causes death (mean survival of about 6 months) (1–4).

Anaplastic thyroid carcinoma treatment includes a multimodal protocol consisting of surgery (5), chemotherapy (doxorubicin and cisplatin), and hyperfractionated accelerated external beam radiotherapy (6) (median patient survival of 10 months) (6).

For these reasons, it could be useful to identificate an effective systemic treatment for ATC, to ameliorate the management of this deadly thyroid cancer (TC) (7).

Aurora kinase inhibitors and tyrosine kinase inhibitors (TKIs) (8), as imatinib (9) or sorafenib (10), are promising future treatments, while other studies (11–15) have evaluated antiangiogenic agents, like PTK787/ZK222584, aplidin, combretastatin A4 phosphate, and human vascular endothelial growth factor (VEGF) monoclonal antibodies (bevacizumab, cetuximab).

Moreover, small-molecule adenosine triphosphate (ATP) competitive inhibitors directed intracellularly at epidermal growth factor receptor (EGFR)'s tyrosine kinase (such as erlotinib, or gefitinib) (16, 17) are under evaluation.

The antitumor activity of CLM94 [a new cyclic amide, with antiangiogenic effect and anti-VEGF receptor (R)-2], has been shown *in vitro* and *in vivo* in primary (p)ATC cells (18), such as a potent antitumor activity of the new “pyrazolo[3,4-d]pyrimidine” compounds (CLM29 and CLM24), with an antiangiogenic action and able to inhibit EGFR, the RET tyrosine kinase, VEGFR, in 8305C and pATC cells (19).

Moreover, CLM3 (with antiangiogenic activity and suggested for a multiple signal transduction inhibition, on EGFR, the RET tyrosine kinase, and VEGFR), has shown antitumor and antiangiogenic activity in pATC cells (20).

Recently, the combination of dabrafenib plus trametinib has been recently approved for the treatment of ATC with ^*V600E*^*BRAF* mutation (21, 22).

Moreover, we have recently shown, that lenvatinib, and vandetanib, have a significant antineoplastic effect, *in vitro* in ATC cells, and in xenotrasplants of ATC *in vivo* in nude mice (23, 24).

Despite these new therapeutic strategies against ATC, more researches are required to identify therapies able to control and to cure this disease.

Testing the sensitivity of pATC cells from each subject to different drugs could give the possibility to increase the effectiveness of the treatment in the next future, for the personalization of the therapy.

By disease-orientated *in vitro* drug testing conducted in human neoplastic cell lines, predictive values for the activity of clinical responses can be obtained (25, 26). A 60% positive predictive value and a 90% negative predictive value have been reported (27). Therefore, *in vitro* drug testing could avoid to administer patients with inactive chemotherapeutics.

Until now, pATC have been obtained after surgery (biop-pATC) for therapeutic or diagnostic techniques. However, it has been shown the possibility to obtain pATC from fine-needle aspiration (FNA), avoiding worthless surgical procedures and allowing the evaluation of the sensitivity to different chemotherapeutic agents in each patient (28–30).

In this study, we evaluate the antineoplastic effect of lenvatinib, and vandetanib, in pATC obtained from biop-pATC, or from FNA-pATC.

## Methods

### Drugs and Supplements

Lenvatinib (E7080, Lenvima; 1 nM, 100 nM, 1, 10, 25, and 50 μM), and vandetanib (ZD6474, Caprelsa; 1 nM, 100 nM, 1, 10, 25, and 50 μM), were evaluated in pATC cell cultures.

Most of chemicals and supplements were obtained from Sigma-Aldrich (Merck KGaA, Darmstadt, Germany).

### Patients Source for Thyroid Tissue

Thyroidal tissues were obtained from 6 patients with ATC at the time of surgery. The diagnosis was done following generally recognized clinical, laboratory, and histological criteria (28–30).

Absence of thyroid-stimulating hormone (TSH) receptor, thyroperoxidase (TPO), thyroglobulin (Tg), and Sodium/Iodide Symporter (NIS) expression has been shown by immunohistochemistry.

Microdissection and DNA extraction, detection of *BRAF* mutation by PCR Single Strand Conformation Polymorphism (PCR-SSCP) and direct DNA sequencing were performed using conventional methods previously described (28–30).

Informed consent to the study was obtained from all the subjects, and the approval was received from the local ethical committee of the University of Pisa.

### Primary ATC Cells

#### FNA-pATC

Fine-needle aspiration was conducted in 6 ATC patients by FNA cytology (23 gauge needle). About 10,000 cells were seeded in RPMI 1640 containing 20 μg/ml gentamicin, 100 IU/ml penicillin G, 1% w/v glutamine, 20% v/v Fetal Calf Serum (FCS) (Seromed, Biochrom, Berlin, Germany). After 2 weeks, cells were propagated in DMEM medium containing 50 μg/ml penicillin/streptomycin, 1% w/v glutamine and 20% v/v FCS, then incubated at 37°C in 5% CO_2_.

To have a sufficient number of cells, chemosensitivity tests were performed at the 4th passage, after 4–5 weeks of controlled *in vitro* growth.

#### Biop-pATC

Neoplastic tissues (1–3 mm in size) were obtained, and washed in M-199 media containing 500 IU/ml penicillin, 500 IU/ml streptomycin, and 1,000 IU/ml nystatin, then suspended in DMEM with 50 μg/ml penicillin/streptomycin, 1% w/v glutamine and 20% v/v FCS and maintained in 5% CO_2_ at 37°C.

At the third cell passage reached in primary tissue-culture flasks, cells were coated in methocel (31) to evaluate the colony-forming efficiencies. The biggest colonies were expanded and chemosensitivity tests were carried out when cells reached the 4th passage.

The absence of TSH receptor, TPO, Tg, and NIS expression was confirmed by immunohistochemistry.

A partial and focal positivity for cytokeratin was obtained by immunocytochemistry on de-stained smears in FNA-pATC.

DNA fingerprinting showed a pattern identical to that of the original neoplastic tissue (28–30).

### WST-1 Assay

Cell viability and proliferation were assessed by the WST-1 assay [3-[4,5-dimethylthiazol-2-yl]-2,5-diphenyltetrazolium bromide, used in the MTT assay, by Roche Diagnostics, Almere, The Netherlands] (28, 29, 32).

Different concentrations of lenvatinib or vandetanib (1 nM, 100 nM, 1, 10, 25, and 50 μM), or their vehicle alone, were added in quadruplicates to cells, that were treated for 24 h. Then IC_50_ values were determined by linear interpolation. The experiments were performed in triplicate for each cell preparation.

For comparison, proliferation was evaluated also by the cell number counting (28, 29, 32).

### Apoptosis Evaluation

ATC cells (35,000 cells/mL) were plated and treated with lenvatinib, or vandetanib, for 48 h in a humidified atmosphere (37°C, 5% CO_2_). Then, pATC were stained with Hoechst 33342, as earlier described (32).

The apoptosis index (ratio between apoptotic and total cells) x100 was calculated.

Moreover, the cells were seeded in Lab-tekII Chamber Slide System (Nalge Nunc International), treated with lenvatinib, or vandetanib, for 48 h, and then treated with Annexin V binding assay (32).

### Data Analysis

Values are given as mean ± SD for normally distributed variables, otherwise as median and [interquartile range]. The experiments were repeated 3 times with the cells from each donor. The mean of the experiments in the 6 specimens from different donors is reported. The mean group values were compared by one-way ANOVA for normally distributed variables, otherwise by the Mann-Whitney *U* or Kruskal-Wallis test. Proportions were compared by the χ^2^ test. *Post-hoc* comparisons on normally distributed variables were carried out using the Bonferroni-Dunn test. Data about apoptosis were analyzed by one-way ANOVA with Newman–Keuls multiple comparisons test.

## Results

### FNA-pATC Cells

#### Viability and Proliferation Assay

**Figure 1:**
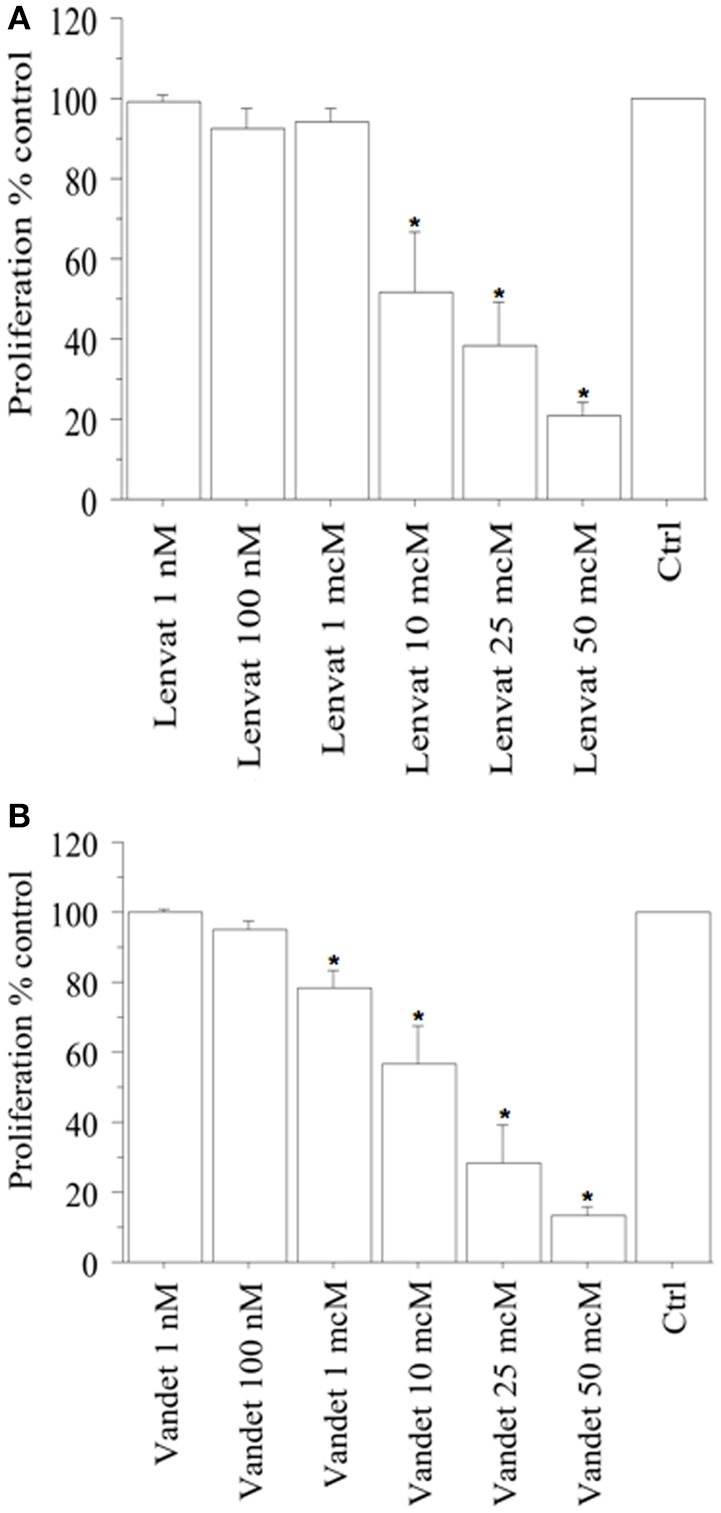
WST-1 test (at 2 h from the beginning of tetrazolium reaction) in FNA-pATC cells treated with lenvatinib **(A)** or vandetanib **(B)** for 24 h. Lenvatinib or vandetanib had a concentration-dependent antiproliferative effect on the FNA-pATC cells with an IC_50_ of 12 or 16 μM, respectively. Bars are mean ± SD. ^*^P < 0.05 or less (by Bonferroni–Dunn test, vs. Control).

In FNA-pATC cells, a significant reduction of proliferation (vs. control) was observed with lenvatinib at 1 h (data not shown) and at 2 h (from the beginning of tetrazolium reaction; *P* < 0.01, for both, ANOVA; Figure 1A), as confirmed by cell counting, too.

In ATC the cell number was 19,405 ± 985/100 μL, per well; 19,589 ± 990 (101%) with lenvatinib 1 nM; 17,850 ± 1,010 (92%) with lenvatinib 100 nM; 18,251 ± 998 (94%) with lenvatinib 1 μM; 10,090 ± 1,115 (52%) with lenvatinib 10 μM; 7,568 ± 1,120 (39%) with lenvatinib 25 μM; 3,687 ± 915 (19%) with lenvatinib 50 μM; (*P* < 0.01, ANOVA). For lenvatinib, IC_50_ was 12 μM (by linear interpolation).

Moreover, also a significant reduction of proliferation (vs. control) was reported with vandetanib at 1 h (data not shown) and at 2 h (from the beginning of tetrazolium reaction; *P* < 0.01, for both, ANOVA; Figure 1B), and confirmed by cell counting.

In ATC the cell number was 19,680 ± 925/100 μL, per well; 19,589 ± 990 (101%) with vandetanib 1 nM; 18,893 ± 995 (96%) with vandetanib 100 nM; 15,744 ± 1,020 (80%) with vandetanib 1 μM; 11,415 ± 1,118 (58%) with vandetanib 10 μM; 5,510 ± 1,120 (28%) with vandetanib 25 μM; 2,755 ± 1,010 (14%) with vandetanib 50 μM; (*P* < 0.01, ANOVA). For vandetanib, IC_50_ was 16 μM (by linear interpolation).

#### BRAF and Proliferation

The ^*V600E*^*BRAF* mutation was present in 2 FNA-pATCs; *RET/PTC1* and *RET/PTC3* by real-time PCR were not revealed in FNA-pATCs.

Regarding the inhibition of proliferation in FNA-pATCs, lenvatinib, and vandetanib gave similar results, considering tumors in presence or absence of the ^V600E^BRAF mutation (data not shown).

#### Apoptosis Determination

**Figure 2:**
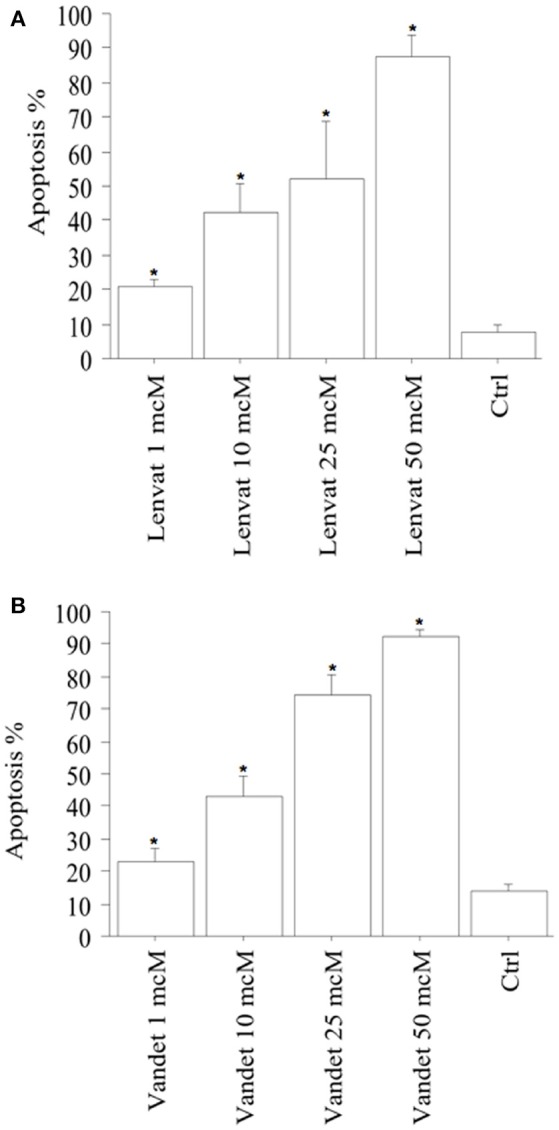
Apoptosis in FNA-pATC cells after the treatment with lenvatinib **(A)** or vandetanib **(B)** for 48 h (mean ± SD of all samples). Apoptosis index was obtained by Hoechst staining. The % of apoptotic cells increased strongly and dose-dependently. Data were analyzed by one-way ANOVA with Newman–Keuls multiple comparisons test and with a test for linear trend (^*^P < 0.001 vs. Control).

Apoptotic cells (expressed in %) in FNA-pATC rised in a dose-dependent manner: 21% of the cells treated with lenvatinib 1 μM were apoptotic; with the higher lenvatinib concentrations of 10 μM, 25 μM or 50 μM the apoptotic percentage increased up to 42, 51, and 88%, respectively (*P* < 0.001, ANOVA; Figure 2A).

Also vandetanib increased apoptosis in FNA-pATC in a dose-dependent manner: 22% of the cells were apoptotic after treatment with vandetanib 1 μM; with the higher vandetanib concentrations of 10, 25, or 50 μM the apoptotic percentage increased up to 42, 72, and 91%, respectively (*P* < 0.001; by ANOVA; Figure 2B). To confirm the induced cell apoptosis, annexin V staining was used (data not shown).

### Biop-ATC Cells

Similar results were obtained in biop-pATC and in FNA-pATC cells, too.

#### Viability and Proliferation Assay

**Figure 3:**
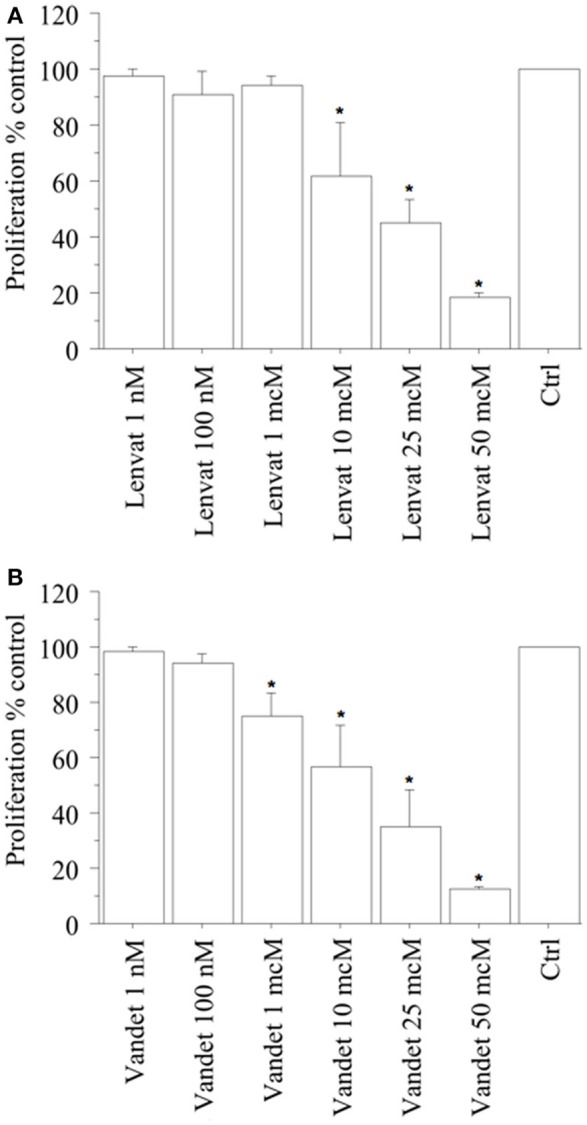
WST-1 test (at 2 h from the beginning of tetrazolium reaction) in biop-pATC cells treated with lenvatinib **(A)** or vandetanib **(B)** for 24 h. Lenvatinib or vandetanib had a concentration-dependent antiproliferative effect on the biop-pATC cells with an IC_50_ of 17 or 18 μM, respectively. Bars are mean ± SD. ^*^P < 0.05 or less (by Bonferroni–Dunn test, vs. Control).

In biop-pATC cells, a significant reduction of proliferation (vs. control) was observed with lenvatinib at 1 h (data not shown) and at 2 h (from the beginning of tetrazolium reaction; *P* < 0.01, for both, ANOVA; Figure 3A), as confirmed by the cell counting.

In ATC the cell number was 19,520 ± 980/100 μL, per well; 19,130 ± 985 (98%) with lenvatinib 1 nM; 17,570 ± 1,132 (90%) with lenvatinib 100 nM; 18,544 ± 996 (95%) with lenvatinib 1 μM; 11,712 ± 11,145 (60%) with lenvatinib 10 μM; 8,589 ± 1,020 (44%) with lenvatinib 25 μM; 4,100 ± 910 (21%) with lenvatinib 50 μM; (*P* < 0.01, ANOVA). For lenvatinib, IC_50_ was 17 μM (by linear interpolation).

Moreover, also a significant reduction of proliferation (vs. control) was reported with vandetanib at 1 h (data not shown) and at 2 h (from the beginning of tetrazolium reaction; *P* < 0.01, for both, ANOVA; Figure 3B), confirmed by cell counting.

In ATC the cell number was 19,270 ± 890/100 μL, per well; 19,070 ± 898 (99%) with vandetanib 1 nM; 18,499 ± 902 (96%) with vandetanib 100 nM; 14,450 ± 998 (75%) with vandetanib 1 μM; 10,984 ± 1,121 (57%) with vandetanib 10 μM; 6,360 ± 1,120 (33%) with vandetanib 25 μM; 2,120 ± 900 (11%) with vandetanib 50 μM; (*P* < 0.01, ANOVA). For vandetanib, IC_50_ was 18 μM (by linear interpolation).

#### BRAF and Proliferation

The ^*V*600*E*^*BRAF* mutation was observed in 2 biop-pATC cells; *RET*/*PTC1* and *RET*/*PTC3* by real-time PCR were not revealed in biop-pATCs.

Considering the inhibition of proliferation in biop-pATCs, lenvatinib, and vandetanib, gave similar results in tumors with/without ^*V*600*E*^*BRAF* mutation (data not shown).

#### Apoptosis Determination

**Figure 4:**
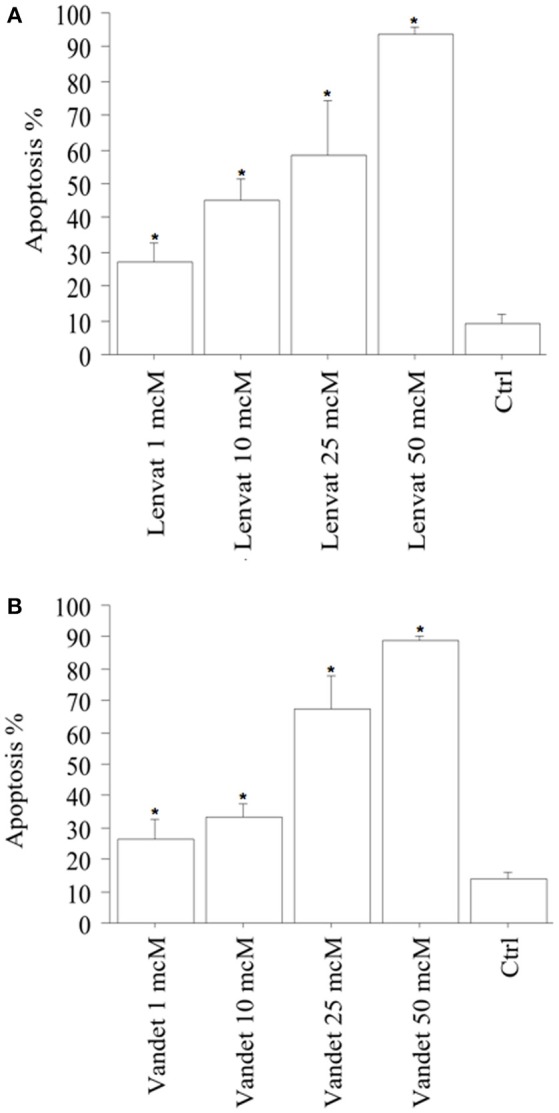
Apoptosis in biop-pATC cells after the treatment with lenvatinib **(A)** or vandetanib **(B)** for 48 h (mean ± SD of all samples). Apoptosis index was obtained by Hoechst staining. The % of apoptotic cells increased strongly and dose-dependently. Data were analyzed by one-way ANOVA with Newman–Keuls multiple comparisons test and with a test for linear trend (^*^P < 0.001 vs. Control).

Apoptotic cells (expressed in %) in biop-pATC cells rised in a dose-dependent manner: 27% of the cells were apoptotic after treatment with lenvatinib 1 μM; with the higher lenvatinib concentrations of 10, 25, or 50 μM the apoptotic percentage increased up to 44, 59, and 92%, respectively (*P* < 0.001, ANOVA; Figure 4A).

Also vandetanib increased apoptosis in biop-pATC dose-dependently: 28% of the cells treated with vandetanib 1 μM were apoptotic; with the higher vandetanib concentrations of 10, 25, or 50 μM the apoptotic percentage increased up to 33, 68, and 89%, respectively (*P* < 0.001; by ANOVA; Figure 4B).

To confirm the induced cell apoptosis, annexin V staining was used (data not shown).

No significant differences in sensitivity to lenvatinib, and vandetanib were observed between the tested cells obtained from FNA or biopsy.

## Discussion

Lenvatinib, and vandetanib are able to exert an antineoplastic action in TC, and ATC. With this study we contribute to understand the lenvatinib, and vandetanib anticancer activity, in ATC, in fact: (1) to the best of our knowledge, this is the first study showing the possibility to screen the antineoplastic activity of lenvatinib, and vandetanib *in vitro* in primary neoplastic cells obtained from cytological samples of FNA; (2) moreover, primary cells from FNA showed a chemosensitivity to TKIs (lenvatinib, and vandetanib) considerably similar to the one in primary cells from biopsy.

Lenvatinib is an oral, multitargeted TKI of VEGFR1-VEGFR3, RET, fibroblast growth factor receptors 1–4 (FGFR1-FGFR4), PDGFRα, and v-kit Hardy-Zuckerman 4 feline sarcoma viral oncogene homolog (KIT) signaling networks involved in tumor angiogenesis (33).

*In vitro* studies evaluated the action of lenvatinib in preclinical models. Lenvatinib had an antineoplastic effect in xenograft models of different cell lines [5 differentiated thyroid cancer (DTC), 5 ATC, and 1 medullary thyroid cancer (MTC)], and had an antiangiogenic effect in 5 DTC and 5 ATC xenografts, while the antiproliferative activity was shown *in vitro* only in 2/11 thyroid cancer cell lines (i.e., RO82-W-1 and TT cells) (34). Moreover, it inhibited RET phosphorylation in TT cells with the activating mutation C634W (34).

*In vivo* phase II (35, 36), and phase III (37) studies in patients with aggressive DTC not responsive to radioiodine showed that lenvatinib administration ameliorated progression-free survival (PFS; median PFS 18.2 vs. 3.6 months with placebo). Following the results of this phase III study, lenvatinib has been approved for the treatment of patients with locally recurrent or metastatic, progressive, radioactive iodine refractory DTC (38).

Other anecdotal studies and a phase II clinical study have an antitumor effect of lenvatinib in ATC (39–43). Furthermore, we have recently reported a significant anticancer activity *in vitro*, and *in vivo*, in experimental models (23, 24).

Vandetanib is an oral once-daily TKI, with a strong antiangiogenic activity, and able to inhibit the activation of RET, EGFR, VEGFR-2, VEGFR-3, and a little of VEGFR-1 (44). A potent antineoplastic action of vandetanib was shown against transplantable MTC in nude mice (45). In patients with aggressive MTC, a phase III clinical study showed vandetanib improved PFS (30.5 vs. 19.3 months in the control group) (46). Food and Drug Administration, and European Medicines Agency approved it in 2011 in patients with locally advanced or metastatic MTC (47) and encouraging data have been shown also in aggressive DTC patients not responsive to the usual therapies (48, 49).

The results of this study agree with the ones of another paper reporting that vandetanib inhibits 8305C cells growth *in vivo*, and stops angiogenesis, decreasing vascular permeability (50), and also with our previous study (24).

Moreover, the results obtained in this study sustain the concept that lenvatinib and vandetanib have antiangiogenesis activity and are suggested for a multiple signal transduction inhibition (on EGFR, the RET tyrosine kinase, VEGFR) (51).

Considering that TKIs inhibitory effects can be bypassed by the activation of other kinases (52), multikinase inhibitors are more useful as they can block more than one single kinase in this way avoiding resistance (53–55).

It is interesting that the anti-proliferative action of lenvatinib and vandetanib did not depend on the presence/absence of ^*V*600*E*^*BRAF* mutation in pATC.

To summarize we can hypothesize that, as shown *in vivo* (23, 24, 56), the antitumor effect of lenvatinib, and vandetanib in the tumoral cells could be linked to the following combination: (1) the antiproliferative action associated with the rise in apoptosis; (2) the inhibition of ERK1/2 phosphorylation (20); (3) the inhibition of tumor neovascularization (18, 56).

By disease-orientated *in vitro* drug testing conducted in human neoplastic cell lines, predictive values for the activity of clinical responses can be obtained (25, 26). A negative predictive value of 90% can avoid to administer patients with inactive chemotherapeutics and a positive preditive value of 60% can predict effectiveness in 60% of cases *in vivo* (27).

The observed disparity between *in vitro* and *in vivo* data can be caused by several factors: the metabolization and/or inactivation of the drugs in the tumor or by different organs in the body (as kidney and liver, etc.); the cellular resistance to drugs; the response to chemotherapeutics that is determined also by the growth curve of tumors (29).

Up to now primary ATC cells have been obtained from surgical materials obtained for therapeutic or diagnostic tecniques. In this study we obtain primary cells from FNA cytology in ATC.

Primary cultures have been obtained by needle aspiration biopsy in only 1 patient (57), and some papers reported of cutaneous needle track seeding after needle aspiration biopsy in TC patients (58, 59), but FNA cytology bypasses this problem and no signs of needle track seeding after FNA has been shown in our patients.

As FNA permits to collect material from a limited area of the tumor, that is the expression of a restricted cell population, this could select a cellular population not representative of the whole tumor. To rule out this possibility, the experiments were repeated with primary cell cultures obtained from bioptical samples in the same conditions. The results were quite similar to those observed in FNA-pATC, in this way excluding the hypothesis that FNA sampling might have brought to a cell population selection.

In conclusion: (1) primary cells obtained from FNA-pATC or biop-pATC, have a similar sensitivity to TKIs; (2) lenvatinib, and vandetanib are are able to decrease cell growth, increasing apoptosis in ATC; (3) the opportunity to test the sensitivity to different TKIs in each patient could avoid to administer ineffective (or even dangerous) drugs to patients, ameliorating also the effectiveness of the therapy; (4) this preclinical evaluation could permit to increase the effectiveness of lenvatinib and vandetanib in patients with ATC in whom the sensitivity has been shown in primary cells *in vitro*.

## Ethics Statement

This study was carried out in accordance with the recommendations of the local ethical committee of the University of Pisa with written informed consent from all subjects. All subjects gave written informed consent in accordance with the Declaration of Helsinki. The protocol was approved by the the local ethical committee of the University of Pisa.

## Author Contributions

SMF, CLM, SU, GM, PF, and AA gave substantial contribution in the conception and design of the work, and in writing the paper. AA and CLM revised it critically for important intellectual content. SMF, CLM, GE, FR, IR, LQ, SRP, SP, AP, SU, EB, GM, PF, and AA gave the final approval of the version to be published and agreed to be accountable for all aspects of the work in ensuring that questions related to the accuracy or integrity of any part of the work are appropriately investigated and resolved.

### Conflict of Interest Statement

The authors declare that the research was conducted in the absence of any commercial or financial relationships that could be construed as a potential conflict of interest.

## Abbreviations

ATC: anaplastic thyroid carcinoma
ATP: adenosine triphosphate
biop-pATC: primary cells from anaplastic thyroid cancer obtained from biopsy
*EGFR*: *epidermal growth factor receptor*
FCS: Fetal Calf Seurum
FNA-pATC: primary cells from anaplastic thyroid cancer obtained from fine needle aspiration
*NIS*: *Sodium/Iodide Symporter*
*pATC*: *primary cells from anaplastic thyroid cancer*
PCR-SSCP: PCR Single Strand Conformation Polymorphism
PFS: progression free survival
TKIs: tyrosine kinase inhibitors
TSH: thyroid-stimulating hormone
TPO: thyroperoxidase
*Tg*: *thyroglobulin*
VEGF: vascular endothelial growth factor
VEGFR: vascular endothelial growth factor receptor.
